# Medical Conditions in Former Professional American-Style Football Players Are Associated With Self-Reported Clinical Features of Traumatic Encephalopathy Syndrome

**DOI:** 10.1089/neur.2024.0008

**Published:** 2024-04-10

**Authors:** Rachel Grashow, Shawn R. Eagle, Douglas P. Terry, Heather DiGregorio, Aaron L. Baggish, Marc G. Weisskopf, Anthony Kontos, David O. Okonkwo, Ross Zafonte

**Affiliations:** ^1^ Harvard Medical School, Boston, Massachusetts, USA.; ^2^ Department of Environmental Health, Harvard T.H. Chan School of Public Health, Boston, Massachusetts, USA.; ^3^ Department of Neurological Surgery, University of Pittsburgh, Pittsburgh, Pennsylvania, USA.; ^4^ Vanderbilt Sports Concussion Center, Department of Neurological Surgery, Vanderbilt University Medical Center, Nashville, Tennessee, USA.; ^5^ Cardiovascular Performance Program, Massachusetts General Hospital, Boston, Massachusetts, USA.; ^6^ Department of Cardiology, Lausanne University Hospital (CHUV) and Institute for Sport Science, University of Lausanne (ISSUL), Lausanne, Switzerland.; ^7^ Department of Orthopedic Surgery, University of Pittsburgh, Pittsburgh, Pennsylvania, USA.; ^8^ Department of Physical Medicine and Rehabilitation, Spaulding Rehabilitation Hospital, Charlestown, Massachusetts, USA.; ^9^ Department of Physical Medicine and Rehabilitation, Harvard Medical School, Boston, Massachusetts, USA.

**Keywords:** chronic traumatic encephalopathy, comorbidities, concussion, encephalopathy syndrome, football, traumatic encephalopathy syndrome

## Abstract

Consensus criteria for traumatic encephalopathy syndrome (TES) specify that at least one core clinical feature of cognitive impairment (CI; e.g., difficulties with memory, executive function) or neurobehavioral dysregulation (ND; e.g., explosiveness, rage, and mood lability) be present and not fully accounted for by other health disorders. Associations between self-reported symptoms that mirror the core clinical features of TES—and how they may be related to concomitant medical conditions—remain unclear. The purpose of this study was to evaluate the association of medical conditions and football exposures with TES clinical features (CI^+/–^, ND^+/–^) in 1741 former professional American-style football (ASF) players (age, 57.7 ± 13.9 years; professional seasons, 6.6 ± 3.9 years). Demographics (age, race/ethnicity, current body mass index, age of first football exposure, use of performance-enhancing drugs, position played, and past concussion symptoms), self-reported medical conditions (anxiety, depression, attention-deficit hyperactivity disorder [ADHD], sleep apnea, headache, stroke, hypertension, heart disease, high cholesterol, erectile dysfunction, and low testosterone) were collected. Of 1741 participants, 7.4% were CI^+^ and/or ND^+^ (*n* = 129). Participants who were CI^+^ or ND^+^ were more likely to report one or more coexisting medical conditions than participants who did not report CI or ND (odds ratio [OR] = 2.04; 95% confidence interval: 1.25–3.47; *p* = 0.003). Separate general linear models for each medical condition that adjusted for demographics and football-related factors identified significant associations between ADHD, diabetes, erectile dysfunction, headaches, sleep apnea, anxiety, and low testosterone and CI^+^ and/or ND^+^ (ORs = 1.8–6.0). Chi-square automatic interaction detection (CHAID) multi-variable decision tree models that incorporated medical conditions and football exposures accurately differentiated former players meeting either CI or ND clinical criteria from those meeting none (accuracy = 91.2–96.6%). CHAID identified combinations of depression, headache, sleep apnea, ADHD, and upper quartiles of concussion symptom history as most predictive of CI^+^ and/or ND^+^ status. CI^+^ and/or ND^+^ players were more likely to report medical conditions known to cause cognitive symptoms. Concussion exposure and medical conditions significantly increased the likelihood that a former ASF player would demonstrate cognitive or neurobehavioral dysfunction. Clinicians engaged with this population should consider whether treatable coexisting condition(s) could account for some portion of the clinical picture associated with TES presentation.

## Introduction

In 2021, Katz and colleagues^1^ published the first research consensus criteria for traumatic encephalopathy syndrome (TES) with funding from the National Institute of Neurological Disorders and Stroke (NINDS). TES was defined as the *in vivo* clinical disorder thought to be associated with a neuropathological diagnosis of chronic traumatic encephalopathy (CTE).^1^ Using a modified Delphi procedure of 20 expert clinician-scientists, consensus was achieved for the research diagnosis of TES that included two core clinical features: 1) cognitive impairment (CI) and/or 2) neurobehavioral dysregulation (ND).^1^ To meet TES criteria, a research participant must also have: 1) history of substantial exposure to repetitive traumatic brain injuries (TBIs); 2) progressive course of the core clinical features; and 3) no other neurological, psychiatric, or medical conditions that can fully account for CI and/or ND clinical features.^1^

Past iterations of TES criteria were not consensus based, and their limitations regarding specificity for predicting CTE neuropathology have been reported. For example, one study evaluated the validity of a past iteration of TES criteria on a sample with donated brains and found a sensitivity of 0.97 and specificity of 0.21.^2^ Notably, the presence of concurrent Alzheimer's disease (AD) in this cohort reduced the odds of an accurate TES diagnosis by 73%. Other studies showed low specificity of past iterations of TES criteria when associated with CTE neuropathological change in former professional football players^3^ and using the consensus definition for TES in high-exposure athletes.^4^ This research highlights the importance of determining whether clinical symptoms of TES could be partially accounted for by other medical conditions that may increase the risk of false-positive TES diagnoses, a condition currently regarded as untreatable.

Previous studies have frequently not reported whether alternative concomitant neurological, psychiatric, or health conditions may account for the cognitive impairment and/or neurobehavioral dysregulation noted in participants. For example, one study showed that 41% of a cohort of 176 professional fighters met clinical criteria for TES, but did not report data related to the fighters' cardiovascular or neuroendocrine medical history.^5^ Another study examined former athletes who met criteria for TES (with and without amyloid-beta neuritic plaques),^4^ but similarly did not report data related to a comprehensive medical history.^6^ As acknowledged by the authors, however, these studies lacked some components of past iterations of the TES criteria.^7,8^ Given that general population studies have documented *perceived* cognitive difficulties and neurobehavioral dysfunction in persons with concurrent psychological (e.g., depression) and cardiovascular disorders (e.g., hypertension), identifying treatable conditions that are associated with TES clinical criteria could offer avenues of symptom reduction while TES treatments are developed.

The purpose of this study was to evaluate associations among demographic factors, health conditions, and football exposures and both CI and/or ND in a large cohort of former professional American-style football (ASF) players. Further clarification of these associations may both improve the specificity of TES criteria and identify potentially treatable alternative underlying conditions among populations with substantial TBI exposure.

## Methods

### Study participants

The FPHS (Football Players Health Study at Harvard University)^9^ recruited a longitudinal epidemiological cohort of former professional ASF players who contracted with any professional football league after 1960.^10^ Of 15,011 participants contacted by electronic or residential mail, 4180 (27.2%) had enrolled as of October 2019 and provided baseline data on demographic characteristics, football-related exposures, and current health status. Of 4180, 47.4% (*n* = 1980) provided follow-up data that included additional football exposures, health status, and measures of current neuropsychological functioning.

Among those with follow-up, 239 participants who reported either AD, vascular dementia, “other” dementia, medication for memory loss, or a CTE diagnosis^11^ were excluded because we could not distinguish cognitive and behavioral symptoms associated with their dementia diagnoses from those associated with a CTE/TES dementia. Retaining these participants in statistical analyses would likely bias the results given how common it is for persons with neurodegenerative disorders to report cognitive and behavioral difficulties. Excluding these participants is a more conservative approach and likely reduced the proportion of the sample who reported cognitive difficulties and emotional dyscontrol—as well as reduced the relationship between these symptoms and other health issues. This study was approved by the institutional review board of the Harvard T.H. Chan School of Public Health, and participants provided informed consent before enrollment.

### Demographic and football measures

Age at follow-up was determined using date of birth and date of survey completion. Race was queried as previously described^12^ and categorized into Black, white, missing and Latino/Asian/Native American/Pacific Islander due to the historically low rates of participation for these groups in ASF.^13^ At baseline, participants self-reported the number of seasons of professional football, age of first exposure to organized football, and field position. To approximate exposure to sports-related concussions accrued during years of active play, we used the concussion symptom score (CSS) whereby participants were asked, “While playing or practicing football, did you experience a blow to the head, neck, or upper body followed by any of the following: headaches, nausea, dizziness, loss of consciousness, memory problems, disorientation, confusion, seizure, visual problems, or feeling unsteady of your feet?” For each of these 10 signs/symptoms, participants chose “none,” “once,” “2 to 5 times,” “6 to 10 times,” or “11 times.” These were coded and summed as previously described^12,14–16^ to create a CSS and categorized into quartiles.

### Current medical conditions

Based on previous research conducted by Grashow and colleagues,^17^ we considered several medical conditions known to be associated with cognitive function. Body mass index (BMI) was calculated from self-reported weight (lbs) and height (inches) as (lbs*0.45/inches*0.025) reported at the time of follow-up. Sleep apnea, stroke, self-reported CTE diagnosis from a medical provider, and dementia were based on a yes/no response to the question: “Has a health care provider ever told you that you have had any of the following diagnoses or health outcomes?” Participants were asked, “Has a medical provider ever recommended or prescribed medication for [condition]” and “Are you currently taking medication [for that condition]?” for the following conditions: attention-deficit hyperactivity disorder (ADHD), anxiety, depression, diabetes, erectile dysfunction, headaches, hypertension, hyperlipidemia, memory loss, and low testosterone. We defined indicators of depression as having a 1) current prescription or prescription recommendation for an antidepressant or 2) total score >3 on the two depression-related questions on the Patient Health Questionnaire (PHQ-4).^11^ We defined indicators of anxiety as having a 1) current prescription or prescription recommendation for an anxiolytic or 2) total score >3 on the two anxiety-related questions on the PHQ-4.^12^

### Clinical symptoms of traumatic encephalopathy syndrome

Eight self-reported cognitive symptoms over the previous week were assessed at both baseline and follow-up using the Quality of Life in Neurological Disorders (Neuro-QoL) Applied Cognition-General Concerns.^18^ Frequency of CI symptoms relating to memory, attention, and concentration was summed and T-scored according to a standardized U.S. sample. As previously described,^14,15^ for the 53 (3.0%) participants missing three or fewer Neuro-QoL items, the missing scores were imputed by taking the mean of all completed items. Participants with four or more missing item responses (*N* = 7) were assigned as “missing.” Emotional dyscontrol and ND over the past week was similarly measured using the Neuro-QoL Emotional and Behavioral Dyscontrol questionnaire,^18^ which asks about the frequency of symptoms including being easily upset, losing one's temper, and being easily irritated. A raw ND score was created by summing the frequency of all eight symptoms, then converted to a T-score. For the 40 (2.3%) participants missing one response on the Neuro-QoL dyscontrol scale, scores from the seven answered questions were averaged and that value was imputed for the missing item. No participants included in the final data set were missing more than one item on the dyscontrol scale.

We applied the core clinical features of the National Institutes of Health (NIH) TES criteria to this sample to the best of our ability given the retrospective nature of data collection for this study. All participants are assumed to meet Criterion I (substantial exposure to repetitive TBIs) given their careers playing football. CI^+^ was operationalized as scoring >1.5 standard deviations (SDs) below the mean on the Neuro-QoL Cognition scale at follow-up (T ≤ 35). Further, to capture the progressive course criterion, participants also had to score >5 T-score points worse on the Neuro-QoL Cognition questionnaire compared to their baseline score (i.e., reduction of 0.5 SDs) to be considered CI^+^. Participants were considered meeting the ND criterion (ND^+^) if they scored ≥1.5 SDs above the mean on the Neuro-QoL Emotional and Behavioral Dyscontrol questionnaire (T ≥ 65). This questionnaire was only given at the follow-up assessment so the progressive course criterion could not be applied to neurobehavioral dysregulation. Participants were considered to have met the core clinical features criterion if they endorsed symptoms consistent with CI and/or ND as aligned with Katz and colleagues.^1^

### Statistical analysis

Univariate differences between participants who met core clinical features and those who did not were assessed across demographic, football-related, and health conditions were determined using Kruskal-Wallis' rank-sum tests for continuous variables and chi-square tests for categorical variables. We ran three separate generalized linear models with a binomial link function to estimate the odds ratio (OR) for meeting 1) the CI feature alone; 2) the ND feature alone; and 3) either CI and/or ND. Separate models included current health conditions (ADHD, anxiety, depression, diabetes, erectile dysfunction, headaches, heart conditions, hypertension, hyperlipidemia, low testosterone, sleep apnea, or stroke) and were adjusted for age, race, BMI, age of first football exposure, main position, concussion symptom score, number of years played, and use of performance-enhancing drugs.

### Chi-square automatic interaction detection analyses

The total data set was randomly partitioned into separate training and testing data sets with a 50% allocation using the Partition node of SPSS Modeler. Three chi-square automatic interaction detection (CHAID) models were built with the training data set to discriminate between the three primary outcomes: 1) CI^+^; 2) ND^+^; and 3) either CI^+^ and/or ND^+^. CHAID builds a decision tree from included variables for discriminating between binary categorical outcomes.^19^ CHAID then evaluates the strength of included variables regarding association to the outcome and iteratively proceeds in developing a decision tree until the inclusion of any other variable does not improve discrimination.^20^ The initial model includes all variables and CHAID agnostically identifies a cut-point for continuous variables and identifies the combination of nominal variables, which maximizes information gain.^21^ Thus, variables that do not significantly contribute to information gain are not retained in the final reported model. Bonferroni's corrections are applied for each variable included in the final model.

Overall accuracy and area under the curve (AUC) are reported for each model. An AUC <0.70 was deemed an unacceptable level of discrimination. Nodes of each decision tree branch were compared with a *post hoc* chi-square test, from which ORs and 95% confidence intervals (95% CI) were reported (*p* < 0.05).^21^ Statistical analyses were conducted using R Language for Statistical Computing^27^ and SPSS Modeler v18.2.2.^22^

## Results

Of all 1980 participants with follow-up data, 239 (12.1%) reported either AD, vascular dementia, “other” dementia, medication for memory loss, or a CTE diagnosis^11^ and were excluded, resulting in a final data set of 1741. Descriptive statistics for 1741 players who were classified as CI^+^ and/or ND^+^ (*n* = 129; 7.4%) and CI^–^/ND^–^ (*n* = 1612; 92.6%) are shown in Table 1. Average age was 57.2 ± 13.9 years. Participants (67.8%) were white, 28% were Black, and the remainder were Latino/Asian/Native American/Pacific Islander (Asian, Pacific Islander, or Native American; *n* = 58; see Table 1) or missing (*n* = 24). Participants reported an average of 6.5 ± 4.0 professional football seasons, an average age of first exposure to organized football of 11.7 ± 3.1 years, and an average current BMI of 30.7 ± 4.8. Approximately 73% of participants reported at least one medical condition (*n* = 1213). Participants who met criteria for either CI or ND were more likely to report one or more medical conditions (OR = 2.04; 95% CI: 1.25–3.47; *p* = 0.003) than participants who did not report CI or ND symptoms. Participants who met criteria for CI and/or ND were ∼7 years younger on average, with a greater proportion of Black former players meeting criteria.

**Table 1.** Cohort Characteristics (Mean [Standard Deviation] or Count [Frequency])

	CI^+^ and/or ND^+^			
	No (*N* = 1612)	Yes (*N* = 129)	Total (*N* = 1741)	*p *value
Age	57.74 (13.91)	50.33 (12.08)	57.20 (13.92)	<0.001
Race (%)				0.011
Black	438 (27.2)	50 (38.8)	488 (28.0)	
Missing	20 (1.2)	0 (0.0)	20 (1.1)	
Latino/Asian/Native American/Pacific Islander	46 (2.9)	6 (4.7)	52 (3.0)	
White	1108 (68.7)	73 (56.6)	1181 (67.8)	
No. of seasons	6.54 (3.95)	6.40 (4.11)	6.53 (3.96)	0.690
Main position (%)				0.345
Defensive back	203 (12.6)	18 (14.0)	221 (12.7)	
Defensive line	160 (9.9)	15 (11.6)	175 (10.1)	
Kicker/punter	62 (3.8)	7 (5.4)	69 (4.0)	
Linebacker	234 (14.5)	19 (14.7)	253 (14.5)	
Offensive line	403 (25.0)	33 (25.6)	436 (25.0)	
Quarterback	90 (5.6)	4 (3.1)	94 (5.4)	
Running back	125 (7.8)	15 (11.6)	140 (8.0)	
Special teams	11 (0.7)	2 (1.6)	13 (0.7)	
Tight end	152 (9.4)	6 (4.7)	158 (9.1)	
Wide receiver	172 (10.7)	10 (7.8)	182 (10.5)	
Age of first exposure	11.76 (3.02)	11.48 (3.52)	11.74 (3.06)	0.334
Performance-enhancing drug use during play (%)	224 (13.9)	26 (20.2)	250 (14.4)	0.051
Concussion symptom score	24.95 (22.45)	42.16 (28.17)	26.24 (23.36)	<0.001
Linemen status (%)	563 (34.9)	48 (37.2)	611 (35.1)	0.601
Pain rating (%)				<0.001
0	74 (4.6)	2 (1.6)	76 (4.4)	
1	159 (9.9)	4 (3.1)	163 (9.4)	
2	273 (16.9)	11 (8.5)	284 (16.3)	
3	286 (17.7)	16 (12.4)	302 (17.3)	
4	259 (16.1)	13 (10.1)	272 (15.6)	
5+	532 (14.6)	81 (15.5)	613 (14.6)	
Missing	29 (1.8)	2 (1.6)	31 (1.8)	
Current BMI	30.59 (4.66)	32.36 (6.09)	30.72 (4.80)	<0.001
Heart condition (%)	263 (16.3)	18 (14.0)	281 (16.1)	0.483
ADHD (%)	71 (4.4)	20 (15.5)	91 (5.2)	<0.001
Headache (%)	66 (4.1)	29 (22.5)	95 (5.5)	<0.001
Anxiety (%)	152 (9.4)	28 (21.7)	180 (10.3)	<0.001
Depression (%)	133 (8.3)	33 (25.6)	166 (9.5)	<0.001
Diabetes (%)	146 (9.1)	21 (16.3)	167 (9.6)	0.007
High cholesterol (%)	523 (32.4)	36 (27.9)	559 (32.1)	0.288
Hypertension (%)	558 (34.6)	43 (33.3)	601 (34.5)	0.768
Stroke (%)	41 (2.5)	2 (1.6)	43 (2.5)	0.484
Sleep apnea (%)	439 (27.2)	54 (41.9)	493 (28.3)	<0.001
Low testosterone (%)	207 (12.8)	28 (21.7)	235 (13.5)	0.005
Erectile dysfunction (%)	339 (21.0)	35 (27.1)	374 (21.5)	0.104

CI, cognitive impairment; ND, neurobehavioral dysregulation; BMI, body mass index; ADHD, attention-deficit hyperactivity disorder.

Medical conditions that were significantly associated with CI^+^ status in adjusted models were ADHD, diabetes, erectile dysfunction, headaches, and sleep apnea, with diabetes and sleep apnea showing the highest ORs (diabetes: OR = 3.509; 95% CI: 1.801, 6.838; *p* < 0.001; sleep apnea: OR = 2.922; 95% CI: 1.596, 5.351; *p* = 0.001; Table 2). The variables that were associated with ND^+^ status were headaches (OR = 6.027; 95% CI: 3.367, 10.788; *p* < 0.001) and depression (OR = 4.334; 95% CI: 2.538, 7.400; *p* < 0.001), followed by ADHD, anxiety, and low testosterone (Table 2). The strongest predictors of having either CI and/or ND were headaches (OR = 4.634; 95% CI: 2.735, 7.850; *p* < 0.001) and depression (OR = 2.911; 95% CI: 1.805, 4.693; *p* < 0.001), with additional significant associations noted with ADHD, anxiety, diabetes, erectile dysfunction, low testosterone, and sleep apnea (Table 2).

**Table 2.** Association of Medical Conditions With Meeting Either Core Consensus Criteria (CI and/or ND) for TES

	CI^+^		ND^+^		CI^+^ and/or ND^+^
Model*^a^*	*N*	OR, [95% CI],* p *value	*N*	OR, [95% CI],* p *value	OR, [95% CI],* p *value
ADHD	1678	2.552, [1.141, 5.711], 0.023	1673	3.323, [1.729, 6.387], 0.000	2.737, [1.513, 4.952], 0.001
Anxiety	1678	1.283, [0.594, 2.769], 0.526	1673	3.093, [1.759, 5.439], 0.000	2.163, [1.308, 3.576], 0.003
Depression	1678	1.700, [0.830, 3.484], 0.147	1673	4.334, [2.538, 7.400], 0.000	2.911, [1.805, 4.693], 0.000
Diabetes	1678	3.509, [1.801, 6.838], 0.000	1673	1.648, [0.814, 3.337], 0.165	2.251, [1.297, 3.907], 0.004
Erectile dysfunction	1678	1.975, [1.040, 3.750], 0.037	1673	1.529, [0.860, 2.720], 0.148	1.782, [1.117, 2.843], 0.015
Headaches	1678	2.350, [1.066, 5.179], 0.034	1673	6.027, [3.367, 10.788], 0.000	4.634, [2.735, 7.850], 0.000
Heart condition	1678	1.029, [0.438, 2.417], 0.948	1673	1.322, [0.633, 2.759], 0.457	1.317, [0.726, 2.388], 0.364
High cholesterol	1678	1.564, [0.851, 2.873], 0.149	1673	0.995, [0.573, 1.726], 0.985	1.165, [0.745, 1.820], 0.504
Hypertension	1678	0.897, [0.475, 1.697], 0.739	1673	1.154, [0.680, 1.958], 0.596	1.098, [0.705, 1.710], 0.679
Low testosterone	1678	1.486, [0.752, 2.938], 0.255	1673	1.890, [1.083, 3.297], 0.025	1.581, [0.974, 2.566], 0.064
Sleep apnea	1678	2.922, [1.596, 5.351], 0.001	1673	1.193, [0.696, 2.045], 0.521	1.955, [1.263, 3.028], 0.003
Stroke	1678	0.593, [0.079, 4.482], 0.613	1673	0.641, [0.144, 2.854], 0.559	0.453, [0.105, 1.961], 0.290

^a^ All models adjusted for age, race, BMI, age of first football exposure, main position, concussion symptom score, number of years played, and use of performance enhancing drugs.

CI, cognitive impairment; ND, neurobehavioral dysregulation; TES, traumatic encephalopathy syndrome; ADHD, attention-deficit hyperactivity disorder; OR, odds ratio; 95% CI, confidence interval; BMI, body mass index.

### Multi-condition phenotypes associated with cognitive impairment criterion

**FIG. 1.**
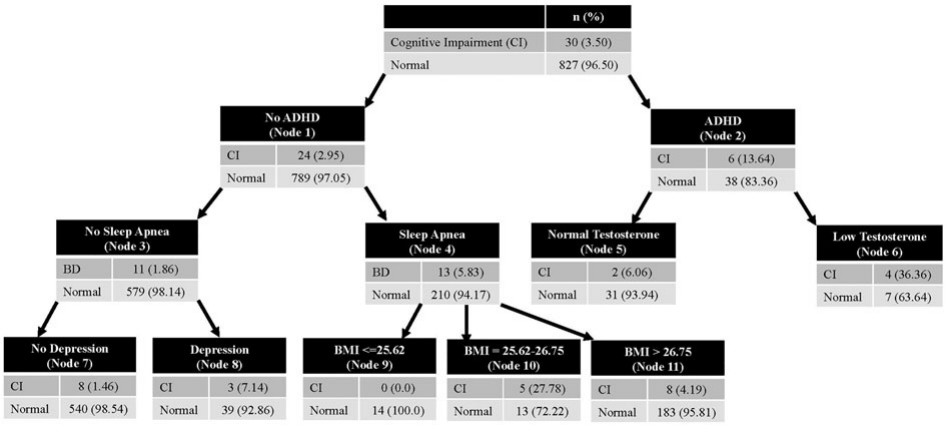
CHAID model differentiating CI^+^ former players from CI^–^ former players. CHAID, chi-square automatic interaction detection; CI, cognitive impairment.

The CHAID model accurately classified players who were CI^+^ in the training (accuracy = 96.5%; *n* = 857) and testing (accuracy = 96.6%; *n* = 890) data sets. AUC was acceptable for the training data set (AUC = 0.74), but not for the testing data set (AUC = 0.59). *Post hoc* associations of medical conditions with the primary outcomes can be viewed in Table 3. Figure 1 shows the decision tree for this model. ADHD was the primary differentiator between groups (OR = 5.19; 95% CI: 1.63–14.03; *p* < 0.001). Among players without ADHD, obstructive sleep apnea (OSA) further discriminated between groups (OR = 3.26; 95% CI: 1.32–8.15; *p* = 0.003). Low testosterone, depression, and BMI were included in the CHAID model, but did not have statistically significant *post hoc* associations with meeting CI criteria.

**Table 3.** Clinical Phenotypes of TES and the Clinical Subcomponents as Determined by the NINDS Consensus Research Criteria

	Phenotype	OR	95% CI
CI^+^	ADHD (Node 2 vs. Node 1)	5.19	1.63–14.03^***^
	No ADHD + OSA (Node 4 vs. Node 3)	3.26	1.32–8.15^**^

ND^+^	Depression (Node 2 vs. Node 1)	7.45	3.72–14.60^***^
	Depression + Headache (Node 6 vs. Node 5)	7.46	1.91–29.06^***^
	Depression + No Headache + Age: ≤48 (Node 12 vs. Node 11)	9.65	1.63–98.71^**^
	No Depression + CSS quartiles = 3–4 (Node 4 vs. Node 3)	9.57	3.25–38.12^***^

CI^+^ and/or ND^+^	Headache (Node 2 vs. Node 1)	11.43	15.13–24.60^***^
	Headache + Depression (Node 6 vs. Node 5)	7.79	1.47–45.19^**^
	No Headache + CSS quartiles = 3–4 (Node 4 vs. Node 3)	3.76	1.87–7.96^***^
	No Headache + CSS quartiles = 1–2 + OSA (Node 8 vs. Node 7)	3.66	1.13–13.44^**^

^***^ *p* < 0.001, ^**^p < 0.01, ^*^p < 0.05.

TES, traumatic encephalopathy syndrome; NINDS, National Institute of Neurological Disorders and Stroke; CI, cognitive impairment; ND, neurobehavioral dysregulation; CSS, concussion symptom score; OSA, obstructive sleep apnea; ADHD, attention-deficit hyperactivity disorder; OR, odds ratio; 95% CI, 95% confidence interval.

### Multi-condition phenotypes associated with neurobehavioral dysregulation criterion

**FIG. 2.**
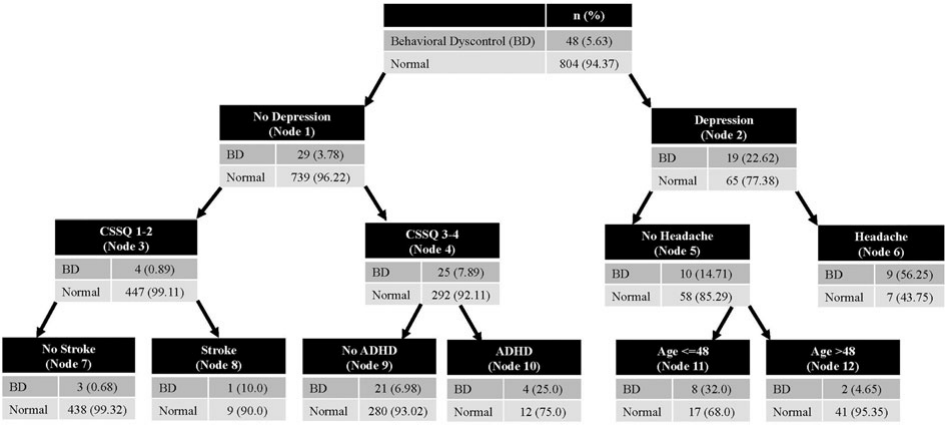
Validated CHAID model differentiating ND^+^ former players from ND^–^ former players. CHAID, chi-square automatic interaction detection; ND, neurobehavioral dysregulation.

The CHAID model accurately classified players who were ND^–^ in the training (accuracy = 94.1%; AUC = 0.83; *n* = 857) data set. This model was validated on the testing data set (accuracy = 94.5%; AUC = 0.71; *n* = 890). Figure 2 shows the decision tree for this model. Depression was the primary differentiator between groups (OR = 7.45; 95% CI: 3.72–14.6; *p* < 0.001). Headache further discriminated between groups among players with depression (OR = 7.46; 95% CI: 1.91–29.06; *p* < 0.001). For players with depression, but not headaches, age (≤48 years) further discriminated between groups (OR = 9.65; 95% CI: 1.63–98.71; *p* = 0.004).

Among players without depression, higher CSS quartiles (3 or 4) discriminated between groups (OR = 9.57; 3.25–38.12; *p* < 0.001). ADHD and stroke were included in the model, but did not have statistically significant *post hoc* associations with meeting the ND criterion.

### Multi-condition phenotypes associated with both core clinical consensus criteria

**FIG. 3.**
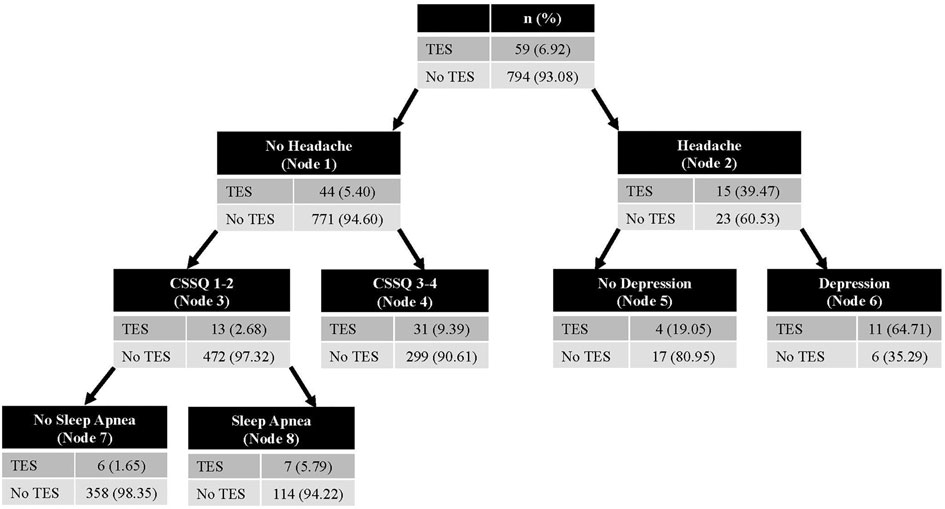
Validated CHAID model differentiating players who were CI^+^ and/or ND^+^ versus players who were CI^–^/ND^–^. CHAID, chi-square automatic interaction detection; CI, cognitive impairment; ND, neurobehavioral dysregulation.

The CHAID model accurately classified players who met either core clinical feature in the training data set (accuracy = 93.2%; AUC = 0.75; *n* = 857) and was validated in the testing data set (accuracy = 91.2%; AUC = 0.73; *n* = 890). Figure 3 shows the decision tree for this model. Headache was the primary differentiator between groups (OR = 11.43; 95% CI: 15.13–24.60; *p* < 0.001). Depression further discriminated between groups among those with headache (OR = 7.79; 95% CI: 1.47–45.19; *p* = 0.007).

Among players without headache, CSS quartiles of 3 or 4 further discriminated between those who had had and did not have any core clinical features (OR = 3.76; 95% CI: 1.87–7.96; *p* < 0.001). OSA further discriminated between players without headache with CSS quartiles of 1 or 2 (OR = 3.66; 95% CI: 1.13–13.44; *p* = 0.015).

## Discussion

In this large cohort study of 1741 former professional American football players, 7.4% self-reported symptoms that correspond with TES core clinical features including CI, ND, or both. Players who met an operationalized definition of clinical TES criteria were twice as likely to report at least one health condition compared to players who did not meet clinical criteria. Former professional football players exhibiting features of CI or ND were more likely to suffer from other medical conditions that produce cognitive or behavioral symptoms, even after adjusting for covariates. Multi-variable decision tree modeling identified clinical phenotypes comprised of health conditions and football exposures that were associated with higher odds of reporting TES symptoms. For example, participants with depression and headaches had a 7.5 times higher likelihood of reporting either of the TES clinical features.

The results of this study suggest that medical conditions (when considered in isolation or in combination) may be associated with a “TES-like” clinical presentation. For example, untreated medical comorbidities that cause cognitive dysfunction, including hypertension,^23^ diabetes,^24^ and hypogonadism^25^ that have been associated with progressive neurocognitive decline could appear to clinically overlap with TES.^12,16^ Complex biological pathways likely underlie associations between exposure to professional ASF play, chronic health conditions, and cognitive symptoms. For example, use of performance-enhancing drugs has been linked to later-life hypogonadism,^26^ which is a risk factor for cognitive dysfunction and mood changes.^27^

Research using past iterations of TES criteria have suggested that they perform with suboptimal diagnostic specificity. The results of the present study suggest that the specificity of the TES consensus criteria represents an area of potential improvement, given that several demographics (e.g., age, race, and higher BMI) and health conditions (e.g., ADHD, anxiety, depression, sleep apnea, low testosterone, diabetes, and headache) were associated with the clinical symptoms included in TES consensus criteria. No study to date has evaluated the validity of the current TES clinical criteria in association with the neuropathological assessment of CTE. In 2021, researchers^2^ applied the 2014 TES criteria^28^ to a cohort of former professional players who donated their brains to a research center. The researchers reported a specificity of 21% for the 2014 TES^28^ criteria identifying neuropathologically confirmed CTE, which was illustrated by no statistically significant difference in core and supportive clinical symptoms (e.g., depression, anxiety, apathy, explosivity, impulsivity, hopelessness, paranoia, suicidality, physical/verbal violence, problem solving, language, fluctuating cognition, and headache) between participants with CTE and those without. Additionally, 85% of the cohort without CTE pathology met TES criteria.^28^

Including medical conditions identified here with future iterations of TES criteria will provide a roadmap for differential diagnosis and clarify the component of TES criteria that requires that clinical presentation not be accounted for by other disorders.

### Limitations

The most notable limitation of this study is the self-reported nature of the outcomes of interest TBI exposure (for which there currently is no established objective measure). Given the nature of this study, it was not feasible to have family or clinic-reported data, which would have offered increased confidence in reported outcomes. As an additional major limitation, cognition was not objectively measured. However, given that subjective symptoms are what drive players to speak to their physicians about cognitive issues, such self-reported clinical symptoms are relevant to understanding manifestations of TES symptoms. We cannot use these data to ascertain whether objective measures of cognitive function would have aligned with those reported on the two Neuro-QOL measures. It is important to note that we used validated measures of subjective cognitive function (Neuro-QOL Applied Cognition-General Concerns and Neuro-QoL Behavioral Dyscontrol), which should create at least a standardized structure for reporting and scoring symptoms.

There are additional limitations related to understanding whether these symptoms are progressive. Cognitive impairment with a progressive course was available through repeated assessments of a subjective cognitive impairment questionnaire. Repeated assessments of the ND survey were not available, so a progressive course could not be demonstrated for dyscontrol. It is possible that an average of 4 years' elapsed time between baseline and follow-up is not sufficient to capture progression; future studies should involve longitudinal studies that extend beyond this time period. Third, we consider potential response bias. The response rate for this study was 27% percent of all invited former players. However, this sample has been shown to be representative of the larger former player population on several key variables such as position, career duration, and age.^9^

There is potential that this sampling method could have attracted the most symptomatic former players. However, this sample also includes former professional football players who signed contracts with a professional league yet were never put on the field during a professional game, thus offering a wider spectrum of exposure to professional football compared with other studies. With an enrollment of >4000 former professional football players, this data set represents the largest study of living former professional players, offering the best representation available to date. This study may not be generalizable to all current professional football players because of potential demographic/sociocultural differences, such that this study included ∼68% white players and the National Football League has had a majority of Black players since the late 1970s.^13^

Last, the analyses presented here cannot determine the causality of medical conditions and/or football exposures on meeting TES consensus criteria. Specifically, the data shown here were used to measure cognitive and behavioral symptoms that may have arisen from neurodegenerative and non-neurodegenerative conditions. Underlying neurodegenerative diseases have also been known to contribute to comorbidities examined in this article, such as anxiety, depression, and sleep problems. This study cannot rule out the possibility that an underlying neurodegenerative condition was present but had yet to be diagnosed. Longitudinal research in this population is necessary to understand the temporal relationship between when diagnosis of medical conditions occur in relation to self-reported TES symptoms and whether additional neurodegenerative conditions are prevalent in this population. Nevertheless, these results underscore the need for clinicians treating these patients to proactively screen for and treat conditions that can be managed while therapeutics are explored to slow or reverse damage occurring as the result of repetitive TBI or connected to CTE.

## Conclusion

In this large cohort study of 1741 former professional ASF players, several medical conditions and higher concussion exposures were associated with higher odds of reporting clinical TES symptoms. It is important for clinicians to screen for and treat these conditions as appropriate, given that many of these conditions are responsive to treatment. Living with untreated conditions may lead the patient and their healthcare team to misattribute symptoms of a medical condition to TES. Because no validated *in vivo* criteria exist, it is impossible to determine whether the clinical phenotypes observed are related to (or co-occur with) existing neuropathology. We encourage researchers studying repetitive TBIs and their potential long-term consequences to assess and consider the effects of these medical conditions on TES criteria and include medical history questionnaires in their studies to rule out other conditions that could account for portions of the participant's clinical presentation. Longitudinal research in at-risk populations is critical to understanding the timeline of medical condition development and football exposures in relation to TES criteria and related health outcomes in post-career players.

## Data Availability

Due to the high profile nature of the participants in this study, data are not available at this time.

## Transparency, Rigor and Reproducibility Statement

This study was not pre-registered online given that data collection has been ongoing for several years. The analysis plan was not pre-registered online, but was conceived and executed by the primary authors and reviewed by two secondary authors with biostatistics training. The primary authors certify that the analysis was pre-specified. The sample size was a convenience sample based upon the response rate of surveys sent out to eligible participants. Data collection and analyses were performed by investigators who were aware of relevant participant characteristics. All outcome measures used are in the public domain. Data and code are not available upon request due to pre-existing agreements with the funding organization that data will not be shared. SPSS v29, SPSS modeler, and R were used to complete the analyses. Missing data were handled as reported in the text. Replication and external validation studies are planned.

## Abbreviations Used

AD: Alzheimer's disease
ADHD: attention-deficit hyperactivity disorder
ASF: American-style football
AUC: area under the curve
BMI: body mass index
CHAID: chi-square automatic interaction detection
CI: cognitive impairment
95% CI: confidence interval
CSS: concussion signs and symptom
CTE: chronic traumatic encephalopathy
ND: neurobehavioral dysregulation
Neuro-QoL: Quality of Life in Neurological Disorders
NIH: National Institutes of Health
NINDS: National Institute of Neurological Disorders and Stroke
OR: odds ratio
OSA: obstructive sleep apnea
PHQ-4: Patient Health Questionnaire
SDs: standard deviations
TBI: traumatic brain injury
TES: traumatic encephalopathy syndrome
